# Rural and Urban Differences in Dementia Diagnosis in the Western U.S.

**DOI:** 10.1093/geroni/igaf122.4256

**Published:** 2025-12-31

**Authors:** Lauren Cater, Andrew Nute, Kara Bensley, Maja Pederson, Brianna Beck, Jessica Fleser, Curtis Noonan, Rachel Peterson

**Affiliations:** University of Montana, Missoula, Montana, United States; Providence Health & Services, Renton, Washington, United States; Providence Health & Services, Renton, Washington, United States; University of Montana, Missoula, Montana, United States; Providence Health & Services, Renton, Washington, United States; Providence Health & Services, Renton, Washington, United States; University of Montana, Missoula, Montana, United States; University of Montana, University of Montana, Montana, United States

## Abstract

Prior research suggests diagnostic prevalence of Alzheimer’s disease and related dementias (ADRD) is lower in rural U.S., though noted geographic variations have been observed in studies of the southern and Midwest regions. We compared rural and urban diagnosed prevalence of ADRD and cognitive impairment in eight Western states: Alaska, California, Idaho, Montana, New Mexico, Oregon, Texas, Washington. Diagnostic codes for ADRD and cognitive impairment from Providence Health system’s electronic medical records were pulled from Jan. 1, 2018-June 30, 2025 for all active patients. Of 6,911,104 patients (min=Idaho: 45,160; max=California: 2.46 million), we observed a substantial difference in overall diagnosed prevalence across states of residence: Oregon and Montana had the highest diagnosed prevalence (6.6% and 6.5%, respectively); New Mexico had the lowest (2.3%). Overall diagnosed prevalence among those residing in urban and high commuting areas (RUCA 1-3, 4.1, 5.1, 8.1, 10.1) was 5.4% versus 4.7% among those residing in rural and low commuting areas (p < 0.001). Within-state urban versus rural differences in diagnosed prevalence was estimated for Alaska (4.8% vs. 3.5%; p < 0.001), California (4.8% vs. 4.2%; p < 0.001), Montana (7.3% urban vs. 5.1% rural; p < 0.001), New Mexico (3.2% vs. 2.2%; p = 0.01), Oregon (6.7% vs. 5.9%; p < 0.001), Texas (3.9% vs. 3.3%; p < 0.001) and Washington (6.3% vs. 5.3%; p < 0.001), but not for Idaho (3.5% vs. 3.4%; p = 0.57). Our findings within a single health system indicate lower ADRD diagnosed prevalence among patients residing in rural areas in 7 of the 8 states examined, suggesting a pattern of lower health system access and diagnosis.

